# Infants' General Movements Were Not Affected by Exposure to Maternal Severe Acute Respiratory Syndrome Coronavirus 2 Infections

**DOI:** 10.1111/apa.70420

**Published:** 2025-12-21

**Authors:** Kathrin Neumayr, Katharina Lippert, A. Sebastian Schroeder, Vinzenz G. Eck, Uta Tacke, Sevil Üzer, Andreas W. Flemmer, Mathias Klemme, Claudia Nussbaum, Nikolas Hesse, Sergi Pujades, Leander Behr, Sonja Strieker, Florian Heinen, Mirjam N. Landgraf

**Affiliations:** ^1^ Department of Paediatric Neurology and Developmental Medicine, iSPZ Hauner MUC, Dr. von Hauner Children's Hospital, LMU University Hospital Ludwig‐Maximilians‐Universität München Munich Germany; ^2^ Center for Child Neurology and Social Pediatrics Child Center Maulbronn Maulbronn Germany; ^3^ Expert Analytics GmbH R&D for Digital Innovations Gauting Germany; ^4^ University Children's Hospital Basel Pediatric Neurology Basel Switzerland; ^5^ Division of Neonatology, Dr. von Hauner Children's Hospital, LMU University Hospital Ludwig‐Maximilians‐Universität München Munich Germany; ^6^ Swiss Children's Rehab and Children's Research Center, University Children's Hospital Zurich Affoltern am Albis Switzerland; ^7^ Inria, Univ. Grenoble Alpes, CNRS, Grenoble INP, LJK Grenoble France; ^8^ School of Computation, Information and Technology Technical University of Munich Munich Germany

**Keywords:** general movement assessment, infant neurodevelopment, neurological disorders, prenatal risk factors, spontaneous motor activity

## Abstract

**Aim:**

Prenatal maternal infections may impair infant brain development. This study investigated the effect of maternal infections with the severe acute respiratory syndrome coronavirus 2 (SARS‐CoV‐2) during pregnancy on infant neurodevelopment by assessing general movements (GMs).

**Methods:**

Infants were recruited for this multicenter prospective cohort study from LMU University Hospital sites in Munich after birth or during routine visits. GMs were recorded and assessed between 1 July 2022 and 31 January 2023, using the Hadders‐Algra method. Odds ratios for abnormal GMs were calculated for infants with parent‐reported prenatal SARS‐CoV‐2 exposure compared to unexposed controls. Specific GM subgroups were analysed at preterm, writhing, and fidgety ages.

**Results:**

The study comprised 114 exposed infants (55.3% male) and 92 controls (54.3% male). There were no significant differences between the groups at birth. Two of the mothers were infected twice and one of them required hospitalisation. GMs were assessed at a mean corrected age (CA) of 4 weeks. The odds ratios showed no significant differences in GM quality between exposed infants and controls in either the total group or the subgroups.

**Conclusion:**

Prenatal exposure to SARS‐CoV‐2 did not affect early neurodevelopment, which was determined by GM quality. Further studies should include long‐term outcomes.

**Trial Registration:**

The study was registered at the German Clinical Trial Register (ID: DRKS00029247; https://drks.de/search/de/trial/DRKS00029247)

## Summary

- This study investigated the effects of the severe acute respiratory syndrome coronavirus 2 (SARS‐CoV‐2) during pregnancy on infant neurodevelopment.
- We assessed the general movements (GMs) of 114 exposed infants and 92 controls from July 2022 to January 2023 using the Hadders‐Algra method, at a mean corrected age of 4 weeks.
- Maternal SARS‐CoV‐2 infections did not increase the odds of abnormal GMs in the total study population or specific GM subgroups.

**Abbreviations**
CA: corrected age
GMA: general movement assessment
GMs: general movements
MOS‐R: motor optimality score—revised
PMA: postmenstrual age
SARS‐CoV‐2: severe acute respiratory syndrome coronavirus 2

## 1 Introduction

Infectious diseases during pregnancy can severely impair neurological outcomes in offspring and require early detection. One suitable, non‐invasive and easily accessible method of evaluating early neuronal integrity is the general movement assessment (GMA).

Maternal immune activation has been associated with adverse neonatal brain development and may increase an infant's risk of developing autism spectrum disorder, schizophrenia or other neurodevelopmental disorders [1, 2]. Maternal viral infections can potentially affect foetal brain development. This is particularly important with regard to the severe acute respiratory syndrome coronavirus 2 (SARS‐CoV‐2), which causes the respiratory disease COVID‐19. Due to its incidence, many pregnant women have been affected by this disease.

Even though the rate of vertical transmission of SARS‐CoV‐2 has been shown to be very low [3], the long‐term effects of prenatal maternal infections on a child's neurological outcomes remain a concern. SARS‐CoV‐2 could lead to a strong maternal immune activation, which might also impact the maternal‐fetal interface [4]. Studies have found associations between COVID‐19 disease in pregnancy and higher rates of preterm birth, stillbirth and preeclampsia [5]. However, studies on the neurodevelopmental sequelae of SARS‐CoV‐2 exposure during fetal life have reported conflicting results. Edlow et al. [6] found a higher rate of neurodevelopmental diagnoses during the first year of life in infants exposed to SARS‐CoV‐2, compared to a non‐exposed control group. In contrast, Ayed et al. [7] reported that 10–12 months after discharge most infants with prenatal SARS‐CoV‐2 exposure showed favourable outcomes, as measured by the Ages and Stages Questionnaire, Third Edition. Conflicting research findings indicate that further studies are needed to monitor the neurodevelopmental outcomes of infants following maternal SARS‐CoV‐2 infections during pregnancy.

One way of evaluating the integrity of the developing nervous system at an early stage is to assess spontaneous, undirected whole‐body movements. These are known as general movements (GMs) and were first described by Heinz Prechtl [8, 9]. GMs can first be observed from 9–10 weeks of postmenstrual age (PMA) and they are then replaced by goal‐directed movements at around 4 months of corrected age (CA) [10, 11]. Their characteristic appearance classifies them as preterm GMs, writhing GMs, and fidgety GMs. Preterm GMs are present from around 28 to around 36–38 weeks of PMA. Writhing GMs appear from around 36–38 weeks of PMA until around 6–8 weeks of CA. Finally, fidgety GMs occur from around 6–8 weeks of CA until around 3–5 months of CA [12, 13]. Two main methods have been established for evaluating GMs, by Prechtl and Hadders‐Algra. While both methods target the same movement qualities [12], a direct comparison is challenging due to their different focuses and classification systems.

The Prechtl method contributed to the development of the Motor Optimality Score—Revised (MOS‐R) [14, 15], a semi‐quantitative method with a 28‐point scale. It focuses on specific movement patterns and fidgety movements [16]. In contrast, the Hadders‐Algra method provides a qualitative assessment that also focuses on milder anomalies. It also evaluates fidgety movements but places less emphasis on them [12]. It has been reported to be a sensitive indicator of general brain function [13]. According to Hadders‐Algra, the GMA involves evaluating the entire movement repertoire and is based on the criteria of complexity, variability, and, to a lesser extent, fluency [12, 13]. The best way to assess whether the infant meets the criteria is to perform the GMA using video data of them moving in an optimal state of active wakefulness [13, 17]. The recorded movements are then classified as normal‐optimal, normal‐suboptimal, mildly abnormal, or definitely abnormal [13].

Both methods have proven to be valuable tools for predicting cerebral palsy [18, 19]. A systematic review showed that the Prechtl method achieved a sensitivity of 97% and a specificity of 89% in predicting cerebral palsy at the fidgety age. Corresponding values at the writhing age were lower. The Hadders‐Algra method showed a sensitivity of 89% and a specificity of 81% at the fidgety age [20].

Predicting neurodevelopmental outcomes, especially the development of cerebral palsy, is more accurate when the GMA is performed at the fidgety age than at an earlier age [12]. However, the sensitivity for detecting cognitive impairment was high when GMs were assessed in the first month of life. Outcomes were measured using the Bayley Scales of Infant and Toddler Development, Third Edition, and the Differential Ability Scale—Second Edition. The sensitivity of GMA reached 80% for outcomes at 2 years and 89% at 4 years [19].

Various studies that used either the Hadders‐Algra or Prechtl methods have also investigated the predictive reliability of GMs beyond cerebral palsy. A population‐based study that used the Hadders‐Algra method at fidgety age found a negative predictive value of nearly 100% for the presence of major neurodevelopmental impairments. These results suggest that the absence of definitely abnormal movements at the fidgety age was associated with a low risk of developing major impairments [18]. In a group of infants born before 33 weeks of gestation, mildly or definitely abnormal GMs, determined by the Hadders‐Algra method, showed a sensitivity of 96.3% in predicting atypical neurological outcomes. This was defined as a cerebral palsy diagnosis or a score of no higher than 70 on the Psychomotor Development Index or Mental Developmental Index of Bayley Scales of Infant Development, Second Edition [21].

Abnormal GMs have been associated with conditions such as attention deficit hyperactivity disorder, behavioural and learning problems [22, 23], and cognitive dysfunctions. These have been measured by the Bayley Scales of Infant and Toddler Development, Third Edition and other tests [24]. Expressive language development has also been linked to GM quality, as smooth and fluent movements have been associated with better outcomes [25].

The GMA has also been used to assess the impact of maternal infections during pregnancy. One study looked at infants who were prenatally exposed to the Zika virus and were not diagnosed with microcephaly. Using the GMA achieved a sensitivity of 70% and a specificity of 96% in predicting adverse neurological outcomes [26].

The available data on the effects of maternal SARS‐CoV‐2 infections on GMs have been very limited. One study by Aldrete‐Cortez et al. [27] used the MOS‐R to assess the early motor repertoire. They found significantly lower scores in 28 infants exposed to SARS‐CoV‐2 than in 28 controls who were not. Similar results were observed by a cohort study that also used the MOS‐R and reported a significantly lower median in 124 prenatally exposed infants. They were compared to data on a pre‐pandemic control group of patients [28].

The aim of this study was to investigate how maternal SARS‐CoV‐2 infections during pregnancy affected infants' general movements, which indicate early neurodevelopmental outcomes.

## 2 Methods

### 2.1 Study Design

This research was part of a multicenter prospective cohort study that assessed infants' general movements.

Infants were enrolled between 1 July 2022 and 31 January 2023 at the Ludwig‐Maximilians‐Universität München in Munich, Germany. We included infants with and without prenatal, perinatal and postnatal risk factors or diagnosed diseases between birth and a corrected age of 20 weeks. The exclusion criteria were infections that required isolation, clinical instability that required life support, or other interventions that would have prevented undisturbed video recording.

### 2.2 Participants

The study population comprised 289 infants recruited at the hospital's study sites shortly after birth or during regular follow‐up examinations at the neuropediatric outpatient centre. This means that infants with a higher risk for neurological impairment were also included. We collected the mother's and child's medical histories and delivery details from hospital records, a questionnaire and an interview. Particular attention was paid to possible SARS‐CoV‐2 infections during pregnancy. The infant was considered to have been prenatally exposed to the virus if the mother stated in the questionnaire or during the interview that she was infected by SARS‐CoV‐2 during pregnancy. Infants were assigned to the control group if their mothers had not had SARS‐CoV‐2 and excluded from analysis if there was no information on maternal infections. Parents were invited to take part in separate optional follow‐up recordings of their infants, which were scheduled at convenient times to improve participation.

### 2.3 Video Recordings and GMA Tests

Hadders‐Algra's qualitative GMA test was used to assess the risk of developing neurological impairments, due to its clinical feasibility, parental acceptance and easy application. It does not attribute the same importance to fidgety movements as Prechtl's method [12] and is more suitable for evaluating the broader age range in our cohort.

The infants' unperturbed movements were filmed using a Kinect camera (Microsoft Corp, Washington, USA) for 3–5 min.

The recordings were assessed by at least two independent, blinded assessors trained in the Hadders‐Algra method, who were doctoral students in medicine, with prior coursework in paediatrics and neurology. They were supervised by paediatricians with many years of clinical experience in neonatology, neuropediatrics and the GMA. The structured, two‐day, self‐assessed training programme used 30 expert‐rated demonstration videos and instructions. The assessors also reviewed additional sample videos for several weeks with an expert panel.

The statistical analysis focused on movements classified as definitely abnormal, mildly abnormal, normal‐suboptimal and normal‐optimal, according to the Hadders‐Algra criteria [13]. These were further subdivided into two categories so that we could transform qualitative categories into dichotomous values that could be used to calculate odds ratios. Definitely abnormal and mildly abnormal were grouped together as abnormal, and normal‐suboptimal and normal‐optimal were considered normal. The scores were included if the assessors agreed on the GM ratings, and any disagreement was discussed by at least four GMA experts until a consensus was reached.

Videos that did not meet the GMA requirements were excluded, such as those where the infant was sleeping or crying.

### 2.4 Statistical Analysis

The statistical analysis was conducted using SPSS software, version 29 (IBM Corp, New York, USA) and the programming language Python, executed in PyCharm 2022.3 Community Edition (JetBrains s.r.o., Prague, Czech Republic). We compared the odds of obtaining abnormal GM scores between the exposed and control groups and tested for significance using the chi‐square test or Fisher's exact test.

The analyses were based on the most recent suitable video. GMs can be classified by their characteristic appearance at three different ages. The study population was divided into preterm, writhing and fidgety ages, based on the age when the infants were recorded. This helped to improve the comparability of the results. Boundaries between the GM categories, especially during the transition between the writhing and fidgety ages, are fluid. Videos of children during this transition period were allocated to the age category that corresponded to the movements they displayed. This meant that each video was only included in the analysis once. If there were multiple recordings during an age group, the most recent was selected. Infants close to the upper age limit of 20 weeks of CA, who no longer showed GMs, were excluded.

**FIGURE 1:**
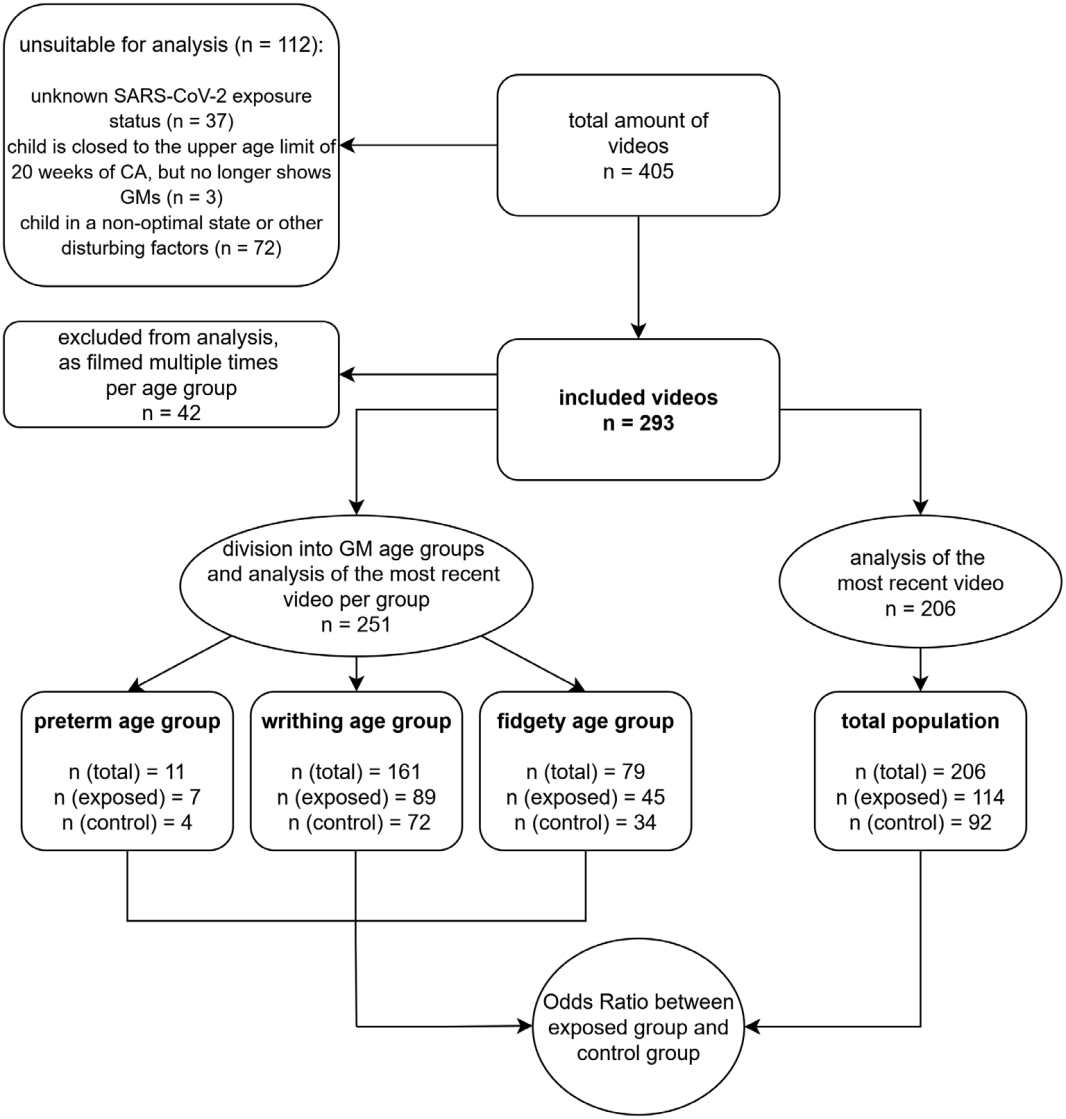
Analytical process. Abbreviations: *n*, number.

The analytical steps are described in Figure 1.

The chi‐square test was used to compare the group characteristics for categorical variables. However, Fisher's exact test was used if the samples were small. The parametric data were tested for normal distribution and variance homogeneity, but these conditions were not fulfilled and the Mann–Whitney U test was performed. The results are shown in Table 1.

**TABLE 1:** Infant and maternal characteristics.

Infant characteristics	Exposed group (*n* = 114)	Control group (*n* = 92)	95% CI for the difference	*p*
Male (*n*; %)	63; 55.3%	50; 54.3%		0.896^##^
Female (*n*; %)	51; 44.7%	42; 45.7%		0.896^##^
Gestational age (mean; SD)	38 weeks + 4 days; 2 weeks + 6 days	38 weeks + 1 day; 2 weeks + 5 days	[−1 week; 0 week]	0.085^++^
Birth weight (mean; SD) *Unknown birth weight (n; %)*	3177 g; 689 g *0; 0%*	3133 g; 654 g *2; 2.2%*	[−210.0; 110.0]	0.495^++^
Birth length (mean; SD) *Unknown birth length (n; %)*	50.1 cm; 4.3 cm *0; 0%*	50.0 cm; 4.3 cm *2; 2.2%*	[−1.0; 1.0]	0.878^++^
Head circumference (mean; SD) *Unknown head circumference (n; %)*	34.6 cm; 3.5 cm *0; 0%*	34.3 cm; 2.2 cm *4; 4.3%*	[−0.5; 0.5]	0.702^++^
APGAR after 1 min (median; IQR) *Unknown APGAR after 1 min (n; %)*	9; 1 *0; 0%*	9; 1 *3; 3.3%*	[0; 0]	0.063^++^
APGAR after 5 min (median; IQR) *Unknown APGAR after 5 min (n; %)*	10; 1 *0; 0%*	10; 1 *3; 3.3%*	[0; 0]	0.364^++^
APGAR after 10 min (median; IQR) *Unknown APGAR after 10 min (n; %)*	10; 0 *0; 0%*	10; 0 *3; 3.3%*	[0; 0]	0.526^++^
Umbilical cord pH (mean; SD) *Unknown pH (n; %)*	7.28; 0.09 *0; 0%*	7.28; 0.08 *4; 4.3%*	[−0.03; 0.02]	0.525^++^
Non‐invasive ventilation (*n*; %) *Unknown non‐invasive ventilation (n; %)*	16; 14.0% *0; 0%*	12; 13.0% *3; 3.3%*		0.910^##^
Invasive ventilation (*n*; %) *Unknown invasive ventilation (n; %)*	1; 0.9% *0; 0%*	3; 3.3% *3; 3.3%*		0.321 ^#^
Maternal characteristics				
Age (mean; SD) *Unknown age (n; %)*	34.6 year; 4.6 year *2; 1.8%*	33.9 years; 5.0 years *4; 3.3%*	[−2.0; 0]	0.524^++^
BMI (mean; SD) *Unknown BMI (n; %)*	23.8; 4.7 *2; 1.8%*	23.0; 4.5 *6; 3.3%*	[−1.730; 0.140]	0.1^++^
Consanguinity of parents (*n*; %) *Unknown consanguinity (n; %)*	2; 1.8% *5; 4.4%*	1; 1.1% *9; 9.8%*		1.0^#^
Alcohol use (*n*; %) *Unknown alcohol use (n; %)*	8; 7.02% *8; 7.0%*	9; 9.78% *8; 8.7%*		0.447^##^
Smoking (*n*; %) *Unknown smoking (n; %)*	3; 2.6% *3; 2.6%*	7; 7.6% *6; 6.5%*		0.107^#^
Medication (*n*; %) *Unknown medication (n; %)*	0; 0% *0; 0%*	2; 2.2% *3; 3.3%*		0.191^#^
Diabetes mellitus (*n*; %) *Unknown diabetes mellitus (n; %)*	6; 5.3% *2; 1.8%*	8; 8.7% *7; 7.6%*		0.273 ^##^
Hypertension (*n*; %) *Unknown hypertension (n; %)*	15; 13.2% *2; 1.8%*	8; 8.7% *7; 7.6%*		0.389^##^
Thyroid disease (*n*; %) *Unknown thyroid disease (n; %)*	36; 31.6% *2; 1.8%*	18; 19.6% *8; 8.7%*		0.097^##^
Other infectious diseases (*n*; %) *Unknown other infectious diseases (n; %)*	8; 7.0% *0; 0%*	9; 9.8% *3; 3.3%*		0.430^##^
Highest educational level				
‐ No schooling (*n*; %)	0; 0%	0; 0%	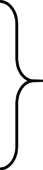	0.005^++^ *
‐ High school graduation/schooling incomplete (*n*; %)	0; 0%	1; 1.1%		
‐ High school graduation/schooling as highest degree (*n*; %)	0; 0%	5; 5.4%		
‐ Vocational college/University degree incomplete (*n*; %)	1; 0.9%	2; 2.2%		
‐ Vocational college/University degree as highest degree (*n*; %)	108; 94.7%	77; 83.7%		
*‐ Unknown educational level (n; %)*	*5; 4.4%*	*7; 7.7%*		

*Note:* ^#^calculated with Fisher's exact test; ^##^calculated with chi‐square test; ^++^calculated with Mann–Whitney *U*‐test.

Abbreviations: BMI, body mass index; CI, confidence interval; IQR, interquartile range; *n*, number; SD, standard deviation.

* *p* ≤ 0.05.

### 2.5 Ethics

The study was approved by the Ludwig‐Maximilians‐Universität München ethics committee (number 454‐16). Written, informed consent was obtained from the infants' parents or legal guardians. Refusal to participate in the study did not affect medical treatment. The infants' faces were blurred before the data were saved and all data were stored pseudonymously.

## 3 Results

### 3.1 Study Sample

We enrolled 289 infants, but 55 were excluded because no suitable video recordings existed and 28 were excluded because their mother's SARS‐CoV‐2 history was unknown. This remaining 206 infants were included in the analysis: 114 (55.3% male) in the exposed group and 92 (54.3% male) in the control group. Of the 114 mothers who reported being infected with SARS‐CoV‐2 during pregnancy, two were infected twice, and one of them required hospitalisation.

Table 1 shows the infants' perinatal characteristics and relevant maternal histories for both groups. The average corrected age at the time of recording was approximately 4 weeks in both groups.

There were no significant differences between the exposed and control groups at birth and both were born at a mean gestational age of 38–39 weeks. Three infants in the exposed group were born extremely preterm before 28 weeks of gestation and 11 were born moderate to late preterm, defined as 32–37 weeks of gestation. Only one control was born extremely preterm, three were born very preterm at 28–31 weeks of gestation, and 11 were born moderate to late preterm.

The only significant difference in maternal history was that educational level was higher in the exposed group (*p* = 0.005).

### 3.2 GMA

It was not possible to follow up all the infants in the three different GM age groups, as this was voluntary and based on the parents' preferences. There were 251 videos that were eligible for analysis: only one infant had videos from all three GM phases, 43 had two, and 162 had one. There were 11 videos in the preterm age group, from seven exposed infants with a mean postmenstrual age of 37 + 2 weeks/days and four controls at 37 + 4 weeks/days of postmenstrual age. We analysed 161 videos at writhing age: 89 from the exposed group at a mean corrected age of 3 days and 72 from the control group at a mean corrected age of 4 days. There were 79 infants in the fidgety age group who were analysed at a mean of 13 weeks of corrected age: 45 from the exposed group and 34 from the control group.

**FIGURE 2:**
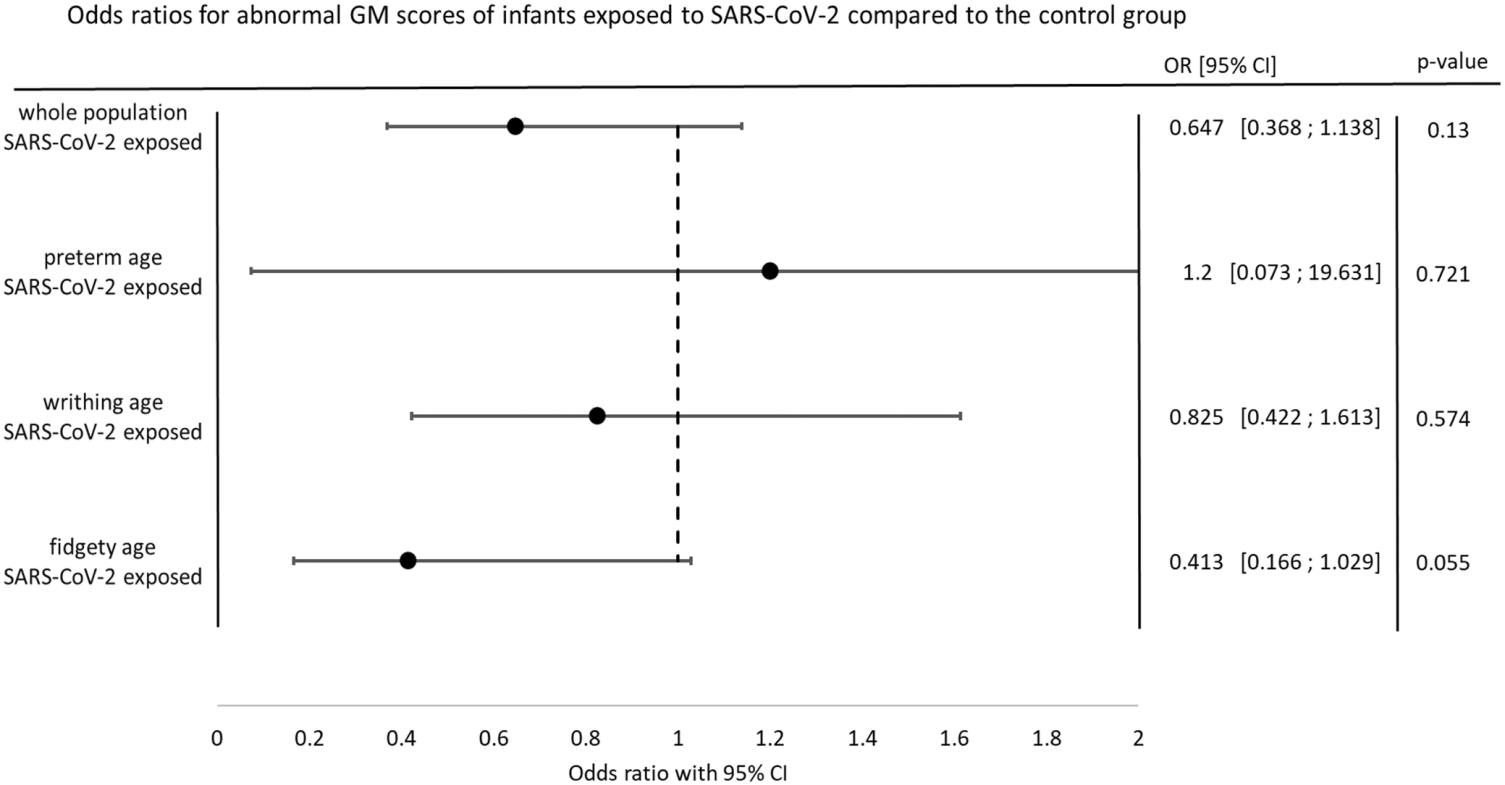
Odds ratios (OR) with confidence intervals (CI) and *p*‐values (significance set at ≤ 5%) for abnormal general movements in infants with prenatal SARS‐CoV‐2 exposure, compared to the unexposed control group in the whole study population as well as in the three age groups (preterm, writhing and fidgety).

Table 2 presents the GM proportions in the entire population and the three age groups and Figure 2 presents the odds ratios for definitely or mildly abnormal GM scores for the exposed and control groups.

**TABLE 2:** Proportion of GM results in the different subgroups.

	GM category	Exposed group	Control group
Whole population	Definitely abnormal	3.5%	8.7%
	Mildly abnormal	30.7%	35.9%
	Normal‐suboptimal	53.5%	45.7%
	Normal‐optimal	12.3%	9.8%
Preterm age	Definitely abnormal	14.3%	25.0%
	Mildly abnormal	14.3%	0.0%
	Normal‐suboptimal	57.1%	50.0%
	Normal‐optimal	14.3%	25.0%
Writhing age	Definitely abnormal	3.4%	5.6%
	Mildly abnormal	25.8%	27.8%
	Normal‐suboptimal	58.4%	56.9%
	Normal‐optimal	12.4%	9.7%
Fidgety age	Definitely abnormal	6.7%	11.8%
	Mildly abnormal	33.3%	50.0%
	Normal‐suboptimal	48.9%	29.4%
	Normal‐optimal	11.1%	8.8%

The analysis did not reveal any statistically significant findings that would indicate a correlation between prenatal exposure to SARS‐CoV‐2 and abnormal GM scores.

## 4 Discussion

This study investigated the impact of prenatal exposure to SARS‐CoV‐2 and did not indicate any general correlation with higher odds of abnormal GM scores in offspring.

These findings did not agree with previous results that suggested lower motor performance at fidgety age in infants prenatally exposed to SARS‐CoV‐2 [27, 28]. The discrepancy between our results and those reported by Aldrete‐Cortez et al. [27] could be attributed to various factors. Their study only focused on 56 infants at fidgety age and included unvaccinated mothers with third‐trimester infections [27]. In addition, the authors used a different GMA method which made a direct comparison challenging. In contrast to the categorial classification of GMs, based on the Hadders‐Algra method, they used the semiquantitative MOS‐R. Both groups showed mildly reduced scores, with a median of 23 points and 21 points in the non‐exposed and exposed groups, respectively [27]. Fajardo Martinez et al. [28] also used the MOS‐R to assess GMs in their cohort study. They reported a significantly lower median MOS‐R of 23 points in infants exposed to SARS‐CoV‐2, compared to 25 points in a pre‐pandemic neurotypical control group. Despite this significant difference, scores above 20 were not considered pathological [28].

Most of the mothers in our study were infected with SARS‐CoV‐2 in the spring and the summer of 2022, when Omicron was the dominant variant, according to the Robert Koch Institute's weekly reports [29]. Studies have linked Omicron to lower hospitalisation rates, milder symptoms, and shorter illness duration than the Delta variant [30]. The possible influence of the different variants on disease severity also complicated how we compared our results with those of pre‐Omicron studies, such as the study by Aldrete‐Cortez et al. [27].

Other studies that used the Ages and Stages Questionnaire reported that SARS‐CoV‐2 did not affect neurological outcomes, which was consistent with our findings. Shuffrey et al. [31] claimed that neither the infection status nor the timing or severity of maternal COVID‐19 disease were associated with infant neurodevelopment at 6 months of age. Similarly, Hessami et al. [32] found that overall neurodevelopment was not affected by in utero exposure to SARS‐CoV‐2.

It was notable that our findings revealed a high prevalence of abnormal GMs in both the exposed and control groups (Table 2). Bouwstra et al. [33] studied a general population cohort at fidgety age and reported 3.7% had definitely abnormal and 25% had mildly abnormal GMs. The corresponding values were slightly higher in our total population. There were 3.5% definitely abnormal and 30.7% mildly abnormal GMs in the exposed group and 8.7% definitely and 35.9% mildly abnormal GMs in the control group. These proportions were even higher when just the fidgety age group was considered. Both the exposed and control groups included infants at increased risk of neurological impairment, who were recruited from study sites that included a university hospital and a neuropediatric outpatient centre. This probably affected the GM quality in our study population and contributed to the increased prevalence of abnormal GMs in both groups. The 56 newborn infants from the general population in hospitals in Mexico City that were studied by Aldrete Cortez al. [27] showed a higher proportion of normal GMs than our study. However, the authors excluded various risk factors, such as birth before 36 weeks of gestation and congenital abnormalities, which we did not. Remarkably, they also reported a non‐optimal performance in the MOS‐R in both the exposed and control groups [27]. Shuffrey et al. [31] reported poorer neurological outcomes in infants born during the pandemic, regardless of SARS‐CoV‐2 exposure. These were reflected by lower scores on subdomains of the Ages and Stages Questionnaires. Pandemic‐related stress factors, such as loneliness and anxiety, may have contributed to the higher prevalence of abnormal GM scores in both groups.

A long‐term follow up is now required to assess the developmental trajectories of infants with abnormal GM scores. A single abnormal score, especially if it is only mildly abnormal, does not necessarily predict poor neurodevelopmental outcomes. Nevertheless, associations with minor neurological dysfunctions have been reported [13].

Studies have shown that differences in birth parameters could potentially influence infants' general movements. These include low gestational age at birth or low birth weight [34], and maternal factors, such as drug use during pregnancy [35]. Therefore, detailed comparisons of the exposed and control groups were performed (Table 1). There were no significant differences, except for lower maternal educational level in the control group. Since mildly abnormal movements have been associated with parental socio‐economic status [33], this factor may have acted as a confounding factor and led to lower GM levels in the control group. This could have masked comparatively lower GM quality in the exposed than the control group.

## 5 Strengths and Limitations

We believe this was the largest study sample to date to compare the association between GM quality and prenatal exposure to SARS‐CoV‐2 with a control group admitted during the same time period. Another strength of our study was that our video material was standardised and recorded in a clinical setting by professionals trained in using the GMA. This ensured high‐quality data and reduced factors that could disturb the infants during the recordings.

However, several limitations should be considered. Dividing GMs according to the three age groups, namely preterm, writhing, and fidgety, may have led to misclassifications, as transitions are fluid. We addressed this problem by asking trained assessors to individually sort the videos into the categories. In addition, this reduced the numbers of infants in the subgroups. For example, there were only 11 infants in the preterm age group and these were challenging to film, as they were born at an early gestational age. This may have limited the statistical power of the results. Whether this specific group was affected by SARS‐CoV‐2 exposure needs to be addressed with larger populations. Such research should also consider the already increased risk of poorer neurodevelopmental outcomes that have been associated with prematurity [36, 37].

Furthermore, separating participants into the exposed and control groups relied solely on maternal reports and we did not know whether they underwent a reverse transcription polymerase chain reaction test, a professionally conducted SARS‐CoV‐2 rapid antigen test, or an antigen self‐test. Consequently, some cases may have been undetected or misclassified. Due to various measures to improve the test coverage in Germany in 2022, we were able to assume that a low number of cases were undetected in our study.

In addition, we did not know the mothers' vaccination status or whether they had a SARS‐CoV‐2 infection before pregnancy. This applied to both groups. We cannot conclude what effect these factors could have had on our cohort.

## 6 Conclusion

The study showed that maternal SARS‐CoV‐2 infections during pregnancy did not increase the odds of abnormal GMs, which point to problems with very early neurological development. Future research should consider the effects that different variants of the virus, maternal vaccination and maternal exposure before pregnancy had on the long‐term neurocognitive development of their offspring.

## Author Contributions

**Kathrin Neumayr:** writing – original draft, formal analysis, investigation, visualization, methodology, validation. **Katharina Lippert:** investigation, methodology, validation, writing – review and editing. **A. Sebastian Schroeder:** conceptualization, project administration, supervision, methodology, validation, writing – review and editing. **Vinzenz G. Eck:** software, data curation. **Uta Tacke:** validation, writing – review and editing. **Sevil Üzer:** writing – review and editing, validation. **Andreas W. Flemmer:** supervision, writing – review and editing. **Mathias Klemme:** resources, writing – review and editing. **Claudia Nussbaum:** resources, writing – review and editing. **Nikolas Hesse:** software. **Sergi Pujades:** software. **Leander Behr:** data curation, formal analysis. **Sonja Strieker:** writing – review and editing. **Florian Heinen:** supervision. **Mirjam N. Landgraf:** conceptualization, project administration, writing – review and editing, supervision, methodology, validation.

## Funding

This study did not receive any specific funding.

## Conflicts of Interest

The authors declare no conflicts of interest.

## Data Availability

Research data are not shared.

## References

[1] D. Elgueta , P. Murgas , E. Riquelme , G. Yang , and G. I. Cancino , “Consequences of Viral Infection and Cytokine Production During Pregnancy on Brain Development in Offspring,” Frontiers in Immunology 13 (2022): 816619, 10.3389/fimmu.2022.816619. 35464419 PMC9021386

[2] B. K. Lee , C. Magnusson , R. M. Gardner , et al., “Maternal Hospitalization With Infection During Pregnancy and Risk of Autism Spectrum Disorders,” Brain, Behavior, and Immunity 44 (2015): 100–105, 10.1016/j.bbi.2014.09.001. 25218900 PMC4418173

[3] I. Barcelos , I. A. A. Penna , A. G. Soligo , Z. B. Costa , and W. P. Martins , “Vertical Transmission of SARS‐CoV‐2: A Systematic Review,” Revista Brasileira de Ginecologia e Obstetrícia 43, no. 3 (2021): 207–215, 10.1055/s-0040-1722256. 33860504 PMC10183886

[4] L. J. Juttukonda , E. M. Wachman , J. Boateng , M. Jain , Y. Benarroch , and E. S. Taglauer , “Decidual Immune Response Following COVID‐19 During Pregnancy Varies by Timing of Maternal SARS‐CoV‐2 Infection,” Journal of Reproductive Immunology 151 (2022): 103501, 10.1016/j.jri.2022.103501. 35231754 PMC8867981

[5] S. Q. Wei , M. Bilodeau‐Bertrand , S. Liu , and N. Auger , “The Impact of COVID‐19 on Pregnancy Outcomes: A Systematic Review and Meta‐Analysis,” CMAJ 193, no. 16 (2021): E540–E548, 10.1503/cmaj.202604. 33741725 PMC8084555

[6] A. G. Edlow , V. M. Castro , L. L. Shook , A. J. Kaimal , and R. H. Perlis , “Neurodevelopmental Outcomes at 1 Year in Infants of Mothers Who Tested Positive for SARS‐CoV‐2 During Pregnancy,” JAMA Network Open 5, no. 6 (2022): e2215787, 10.1001/jamanetworkopen.2022.15787. 35679048 PMC9185175

[7] M. Ayed , A. Embaireeg , M. Kartam , et al., “Neurodevelopmental Outcomes of Infants Born to Mothers With SARS‐CoV‐2 Infections During Pregnancy: A National Prospective Study in Kuwait,” BMC Pediatrics 22, no. 1 (2022): 319, 10.1186/s12887-022-03359-2. 35637442 PMC9149327

[8] H. F. Prechtl and B. Hopkins , “Developmental Transformations of Spontaneous Movements in Early Infancy,” Early Human Development 14 (1986): 233–238, 10.1016/0378-3782(86)90184-2. 3803269

[9] H. F. R. Prechtl , “Qualitative Changes of Spontaneous Movements in Fetus and Preterm Infant Are a Marker of Neurological Dysfunction,” Early Human Development 23, no. 3 (1990): 151–158, 10.1016/0378-3782(90)90011-7. 2253578

[10] A. B. Lüchinger , M. Hadders‐Algra , C. M. van Kan , and J. I. de Vries , “Fetal Onset of General Movements,” Pediatric Research 63, no. 2 (2008): 191–195, 10.1203/PDR.0b013e31815ed03e. 18091359

[11] M. Hadders‐Algra , “Putative Neural Substrate of Normal and Abnormal General Movements,” Neuroscience and Biobehavioral Reviews 31, no. 8 (2007): 1181–1190, 10.1016/j.neubiorev.2007.04.009. 17568672

[12] M. Hadders‐Algra , “Neural Substrate and Clinical Significance of General Movements: An Update,” Developmental Medicine and Child Neurology 60, no. 1 (2018): 39–46, 10.1111/dmcn.13540. 28832987

[13] M. Hadders‐Algra , “General Movements: A Window for Early Identification of Children at High Risk for Developmental Disorders,” Journal of Pediatrics 145, no. 2 (2004): S12–S18, 10.1016/j.jpeds.2004.05.017. 15292882

[14] C. Einspieler , P. B. Marschik , J. Pansy , et al., “The General Movement Optimality Score: A Detailed Assessment of General Movements During Preterm and Term Age,” Developmental Medicine and Child Neurology 58, no. 4 (2016): 361–368, 10.1111/dmcn.12923. 26365130 PMC5951275

[15] C. Einspieler , A. F. Bos , M. Krieber‐Tomantschger , et al., “Cerebral Palsy: Early Markers of Clinical Phenotype and Functional Outcome,” Journal of Clinical Medicine 8, no. 10 (2019): 101616, 10.3390/jcm8101616. PMC6833082 31590221

[16] C. Einspieler and H. F. Prechtl , “Prechtl's Assessment of General Movements: A Diagnostic Tool for the Functional Assessment of the Young Nervous System,” Mental Retardation and Developmental Disabilities Research Reviews 11, no. 1 (2005): 61–67, 10.1002/mrdd.20051. 15856440

[17] M. Hadders‐Algra , Y. Nakae , L. A. Van Eykern , A. W. den Klip‐Van Nieuwendijk , and H. F. Prechtl , “The Effect of Behavioural State on General Movements in Healthy Full‐Term Newborns. A Polymyographic Study,” Early Human Development 35, no. 1 (1993): 63–79, 10.1016/0378-3782(93)90140-p. 8293719

[18] H. Bouwstra , G. R. Dijk‐Stigter , H. M. Grooten , et al., “Predictive Value of Definitely Abnormal General Movements in the General Population,” Developmental Medicine and Child Neurology 52, no. 5 (2010): 456–461, 10.1111/j.1469-8749.2009.03529.x. 20002118

[19] A. J. Spittle , M. M. Spencer‐Smith , J. L. Cheong , et al., “General Movements in Very Preterm Children and Neurodevelopment at 2 and 4 Years,” Pediatrics 132, no. 2 (2013): e452–e458, 10.1542/peds.2013-0177. 23878041

[20] A. K. L. Kwong , T. L. Fitzgerald , L. W. Doyle , J. L. Y. Cheong , and A. J. Spittle , “Predictive Validity of Spontaneous Early Infant Movement for Later Cerebral Palsy: A Systematic Review,” Developmental Medicine and Child Neurology 60, no. 5 (2018): 480–489, 10.1111/dmcn.13697. 29468662

[21] F. de Bock , H. Will , U. Behrenbeck , M. N. Jarczok , M. Hadders‐Algra , and H. Philippi , “Predictive Value of General Movement Assessment for Preterm Infants' Development at 2 Years – Implementation in Clinical Routine in a Non‐Academic Setting,” Research in Developmental Disabilities 62 (2017): 69–80, 10.1016/j.ridd.2017.01.012. 28113095

[22] P. A. van Iersel , S. C. Bakker , A. J. Jonker , and M. Hadders‐Algra , “Does General Movements Quality in Term Infants Predict Cerebral Palsy and Milder Forms of Limited Mobility at 6 Years?,” Developmental Medicine and Child Neurology 58, no. 12 (2016): 1310–1316, 10.1111/dmcn.13228. 27521054

[23] M. Hadders‐Algra and A. M. Groothuis , “Quality of General Movements in Infancy Is Related to Neurological Dysfunction, ADHD, and Aggressive Behaviour,” Developmental Medicine and Child Neurology 41, no. 6 (1999): 381–391, 10.1017/s0012162299000845. 10400172

[24] C. Einspieler , A. F. Bos , M. E. Libertus , and P. B. Marschik , “The General Movement Assessment Helps us to Identify Preterm Infants at Risk for Cognitive Dysfunction,” Frontiers in Psychology 7 (2016): 406, 10.3389/fpsyg.2016.00406. 27047429 PMC4801883

[25] S. Salavati , C. Einspieler , G. Vagelli , et al., “The Association Between the Early Motor Repertoire and Language Development in Term Children Born After Normal Pregnancy,” Early Human Development 111 (2017): 30–35, 10.1016/j.earlhumdev.2017.05.006. 28549271 PMC5951278

[26] C. Einspieler , F. Utsch , P. Brasil , et al., “Association of Infants Exposed to Prenatal Zika Virus Infection With Their Clinical, Neurologic, and Developmental Status Evaluated via the General Movement Assessment Tool,” JAMA Network Open 2, no. 1 (2019): e187235, 10.1001/jamanetworkopen.2018.7235. 30657537 PMC6431234

[27] V. Aldrete‐Cortez , L. Bobadilla , S. A. Tafoya , et al., “Infants Prenatally Exposed to SARS‐CoV‐2 Show the Absence of Fidgety Movements and Are at Higher Risk for Neurological Disorders: A Comparative Study,” PLoS One 17, no. 5 (2022): e0267575, 10.1371/journal.pone.0267575. 35507630 PMC9067650

[28] V. Fajardo Martinez , D. Zhang , S. Paiola , et al., “Neuromotor Repertoires in Infants Exposed to Maternal COVID‐19 During Pregnancy: A Cohort Study,” BMJ Open 13, no. 1 (2023): e069194, 10.1136/bmjopen-2022-069194. PMC9871864 36690405

[29] RKI , “Wochenberichte zu COVID‐19 (donnerstags),” (2023), accessed May 5, 2023, https://www.rki.de/DE/Content/InfAZ/N/Neuartiges_Coronavirus/Situationsberichte/Wochenbericht/Wochenberichte_Tab.html?nn=2444038&cms_gtp=16396118_list%253D2.

[30] C. Menni , A. M. Valdes , L. Polidori , et al., “Symptom Prevalence, Duration, and Risk of Hospital Admission in Individuals Infected With SARS‐CoV‐2 During Periods of Omicron and Delta Variant Dominance: A Prospective Observational Study From the ZOE COVID Study,” Lancet 399, no. 10335 (2022): 1618–1624, 10.1016/s0140-6736(22)00327-0. 35397851 PMC8989396

[31] L. C. Shuffrey , M. R. Firestein , M. H. Kyle , et al., “Association of Birth During the COVID‐19 Pandemic With Neurodevelopmental Status at 6 Months in Infants With and Without In Utero Exposure to Maternal SARS‐CoV‐2 Infection,” JAMA Pediatrics 176, no. 6 (2022): e215563, 10.1001/jamapediatrics.2021.5563. 34982107 PMC8728661

[32] K. Hessami , A. H. Norooznezhad , S. Monteiro , et al., “COVID‐19 Pandemic and Infant Neurodevelopmental Impairment: A Systematic Review and Meta‐Analysis,” JAMA Network Open 5, no. 10 (2022): e2238941, 10.1001/jamanetworkopen.2022.38941. 36306133 PMC9617178

[33] H. Bouwstra , G. R. Dijk‐Stigter , H. M. Grooten , et al., “Prevalence of Abnormal General Movements in Three‐Month‐Old Infants,” Early Human Development 85, no. 6 (2009): 399–403, 10.1016/j.earlhumdev.2009.01.003. 19211203

[34] C. J. Alonzo , L. C. Letzkus , E. A. Connaughton , N. L. Kelly , J. A. Michel , and S. A. Zanelli , “High Prevalence of Abnormal General Movements in Hospitalized Very Low Birth Weight Infants,” American Journal of Perinatology 29, no. 14 (2022): 1541–1547, 10.1055/s-0041-1722943. 33535241

[35] T. Fjørtoft , M. Brandal , A. M. Brubakk , et al., “Maternal Alcohol and Drug Use During Pregnancy Affects the Motor Behaviour and General Movements of Infants Aged 3‐4 Months,” Early Human Development 151 (2020): 105171, 10.1016/j.earlhumdev.2020.105171. 32977207

[36] E. Hee Chung , J. Chou , and K. A. Brown , “Neurodevelopmental Outcomes of Preterm Infants: A Recent Literature Review,” Translational Pediatrics 9, no. 1 (2020): S3–s8, 10.21037/tp.2019.09.10. 32206579 PMC7082240

[37] A. Mitha , R. Chen , N. Razaz , et al., “Neurological Development in Children Born Moderately or Late Preterm: National Cohort Study,” BMJ 384 (2024): e075630, 10.1136/bmj-2023-075630. 38267070 PMC11957549

